# Evaluation of compressed sensing MRI for accelerated bowel motility imaging

**DOI:** 10.1186/s41747-018-0079-9

**Published:** 2019-02-06

**Authors:** C. S. de Jonge, B. F. Coolen, E. S. Peper, A. G. Motaal, C. Y. Nio, I. Somers, G. J. Strijkers, J. Stoker, A. J. Nederveen

**Affiliations:** 1 Department of Radiology and Nuclear Medicine, Amsterdam UMC, location Academic Medical Center, University of Amsterdam, Amsterdam, The Netherlands; 2 Department of Biomedical Engineering and Physics, Amsterdam UMC, location Academic Medical Center, University of Amsterdam, Amsterdam, The Netherlands

**Keywords:** Abdomen, Artifacts, Intestine (small), Magnetic resonance imaging, Magnetic resonance imaging (Cine), Gastrointestinal motility

## Abstract

**Background:**

To investigate the feasibility of compressed sensing and parallel imaging (CS-PI)-accelerated bowel motility magnetic resonance imaging (MRI) and to compare its image quality and diagnostic quality to conventional sensitivity encoding (SENSE) accelerated scans.

**Methods:**

Bowel MRI was performed in six volunteers using a three-dimensional balanced fast field-echo sequence. Static scans were performed after the administration of a spasmolytic agent to prevent bowel motion artefacts. Fully sampled reference scans and multiple prospectively 3× to 7× undersampled CS-PI and SENSE scans were acquired. Additionally, fully sampled CS-PI and SENSE scans were retrospectively undersampled and reconstructed. Dynamic scans were performed using 5× to 7× accelerated scans in the presence of bowel motion. Retrospectively, undersampled scans were compared to fully sampled scans using structural similarity indices. All reconstructions were visually assessed for image quality and diagnostic quality by two radiologists.

**Results:**

For static imaging, the performance of CS-PI was lower than that of fully sampled and SENSE scans: the diagnostic quality was assessed as adequate or good for 100% of fully sampled scans, 95% of SENSE, but only for 55% of CS-PI scans. For dynamic imaging, CS-PI image quality was scored similar to SENSE at high acceleration. Diagnostic quality of all scans was scored as adequate or good; 55% of CS-PI and 83% of SENSE scans were scored as good.

**Conclusion:**

Compared to SENSE, current implementation of CS-PI performed less or equally good in terms of image quality and diagnostic quality. CS-PI did not show advantages over SENSE for three-dimensional bowel motility imaging.

## Key points

- For 3D static bowel imaging, sensitivity encoding (SENSE) sequences outperformed compressed sensing and parallel imaging (CS-PI) sequences.
- The performance of CS-PI increased for 3D dynamic bowel motility scans in comparison to static scans.
- 3D bowel motility imaging did not benefit from CS-PI.

## Background

Intestinal motility frequently becomes deranged in diseases such as Crohn’s disease and Chronic Intestinal Pseudo-Obstruction (CIPO). The resulting hypo-motility in Crohn’s disease and the global pan-enteric changes as seen in chronic intestinal pseudo-obstruction are considered powerful biomarkers to monitor the disease process [1–6]. Unfortunately, existing motility measurement techniques like manometry, capsule endoscopy, and transit time imaging are invasive approaches; they can only be utilised in specialised centres and have technique-specific constraints limiting their clinical use, especially in case of the small bowel.

To overcome the abovementioned problems, magnetic resonance imaging (MRI) has been introduced as a non-invasive technique to assess bowel motility. Promising results have been obtained using dynamic MRI sequences that capture bowel movements between multiple time frames, in combination with sophisticated motion analysis software [2–7]. However, despite these advances, technological challenges with respect to image acquisition remain.

MRI studies on bowel dysmotility have mainly used two-dimensional (2D) techniques. Unfortunately, through-plane movement of bowel loops can easily be obscured by a slice thickness larger than 5 mm [2–5]. Furthermore, previous studies showed that the frequency of small bowel contractions ranges from nine to twelve cycles per minute [1], indicating that a temporal resolution of 2.5 s is required. Finally, for optimal assessment of motility parameters, full coverage of the bowel is desirable. Altogether, this strongly suggests the need for fast three-dimensional (3D) isotropic imaging protocols.

While 3D sequences are beneficial in terms of signal-to-noise efficiency, they are inherently slow compared to 2D sequences because of the need of phase encoding in two directions. Fortunately, advances in accelerated imaging have allowed substantial decreases in acquisition time by reconstruction algorithms that provide diagnostically useful images even from highly undersampled data [8, 9]. State-of-the-art MRI scanners already use parallel imaging (PI) acceleration techniques, such as *sensitivity encoding* (SENSE) [8] or *generalised autocalibrating partial parallel acquisition* [9], which exploit the spatial distribution of multiple coil elements to decrease the number of required k-lines for image reconstruction. Depending on the coil geometry, acceleration factors of 2× to 3× in a single-phase direction are feasible in a clinical setting.

A different acceleration technique that is finding its way into clinical research is *compressed sensing* (CS). This is a method based on iterative reconstruction, which uses the assumption of image sparsity to minimise artefacts from randomly undersampled data [10]. This technique has proven successful in a wide range of MRI applications [11–14], including abdominal imaging, thereby quickly gaining popularity in clinical research as well. Recently, advantages of both CS and PI techniques have been combined into the so-called CS-PI reconstruction algorithms [15]. Therefore, bowel motility measurements might benefit from this technique to achieve the temporal resolution and coverage required in clinic.

The purpose of this study was therefore to investigate the feasibility of CS-PI accelerated bowel motility imaging and to compare its image quality to that of clinically available SENSE accelerated scans.

## Methods

To assess the achievable acceleration factor of CS-PI for bowel motility imaging, resulting scans should be compared to fully sampled reference scans. While this is straightforward in many applications concerning static tissues or during breath-hold (*e.g.*, in the liver), in the bowel, this is complicated by its constant motion. To objectively investigate the use of CS, we have performed static scans after temporarily stabilising bowel motility by administering a spasmolytic drug. This allowed acquisition of fully sampled 3D scans uncorrupted by bowel motion, which served as reference static scans. In an additional session, to capture bowel motility, we acquired CS-PI- and SENSE-accelerated dynamic scans of the small bowel without administering any spasmolytic drugs.

### Volunteers

Six healthy subjects (four males, two females; median age 27.5, range 19–30 years) were recruited prospectively by advertisement: three subjects for static scans and three subjects for dynamic scans. Inclusion criteria included healthy, human volunteers who were willing to undergo minimal bowel preparation and MRI. Exclusion criteria for this study were age younger than 18 years or older than 45 years, history of abdominal surgery, gastrointestinal diseases, or current gastrointestinal symptoms. Additional exclusion criteria were contraindications to MRI and, for the static experiment, contraindications for the spasmolytic drug used. Permission of the Medical Ethics Committee of the Amsterdam UMC was obtained, and all subjects gave full written informed consent.

To ensure a similar preparation among subjects, all volunteers were instructed to fast for 4 h prior to the MRI session. During the 30 min prior to the MRI scans, they ingested 1 L of 2.5% mannitol solution at regular intervals of 10 min. Mannitol served the purpose of good bowel distension and contrast for clinical evaluation. To secure minimal movement of the bowel, which inevitably causes severe motion artefacts in the fully sampled scan, 10 mg of scopolaminebutyl (Buscopan; Boehringer-Ingelheim, Ingelheim, Germany) was intravenously injected directly before the fully sampled non-accelerated scan was acquired in the static session.

### General MRI protocol

Scans were acquired in supine position with a 3-T Ingenia scanner (Philips, Best, The Netherlands), using a combination of a posterior coil located in the table (number of channels = 16) and an anterior torso-coil (number of channels = 16) covering the entire abdominal region. After initial scout sequences, coronal 3D scans of the bowel were acquired in multiple breath-holds using a balanced fast field-echo sequence. This sequence was chosen because of its superior lumen wall contrast, a key component for existing tracking techniques for quantification of small bowel motility [2–4]. The scan parameters were chosen based on the settings of the diagnostic abdominal balanced fast field-echo sequences at our centre (Table 1).

**Table 1:** Scan parameters of the fully sampled sequence

Sequence type	Three-dimensional balanced fast field-echo
Field of view	400 × 400 × 100 mm^3^
Repetition time / echo time	2.5/1.26 ms
Voxel size	2.5 × 2.5 × 2.5 mm^3^
Flip angle	20°
Matrix	160 × 159 × 40
Reconstruction voxels	1.39 × 1.39 × 2.5 mm^3^
Sensitivity encoding / Half scan / Keyhole	No
Scan duration	16.4 s

### Experiments

Two experiments were performed: a static experiment and a dynamic experiment. In the static experiment, fully sampled reference scans were acquired with an acquisition time of 16.4 s, followed by multiple prospectively undersampled CS-PI and SENSE scans using acceleration factors of 3×, 4×, 5×, 6×, 7×. Thus, in total, eleven scans per volunteer were acquired. In the dynamic experiment, multiple prospectively undersampled CS-PI and SENSE scans were acquired using acceleration factors of 5×, 6×, 7×, acquiring 6, 8, 9 time frames, respectively (nine scans per volunteer in total). The static and dynamic scan protocols are listed in Table 2.

**Table 2:** MRI protocols and acquisition order of the sequences

Static protocol	Dynamic protocol
1. Survey	1. Survey
2. Administration of 10 mg scopolaminebutyl	2. CS-PI 1 5×
3. Fully sampled sequence as in Table 1	3. CS-PI 2 5×
4. CS-PI 3×	4. SENSE 5×
5. SENSE 3×	5. CS-PI 1 6×
6. CS-PI 4×	6. CS-PI 2 6×
7. SENSE 4×	7. SENSE 6×
8. CS-PI 5×	8. CS-PI 1 7×
9. SENSE 5×	9. CS-PI 2 7×
10. CS-PI 6×	10. SENSE 7×
11. SENSE 6×	
12. CS-PI 7×	
13. SENSE 7×	

*CS-PI* compressed sensing and parallel imaging, *SENSE* sensitivity encoding

### Undersampling strategies

An in-house developed scanner software patch (PROUD, or PROspective Undersampling in multiple Dimensions) was implemented to sample predefined k-space trajectories. These were read from a text-file created in MATLAB (The Mathworks Inc., NA, MA, USA), containing all subsequent (k-*y*, k-*z*)-coordinates.

#### Static scan sessions

**Fig. 1:**
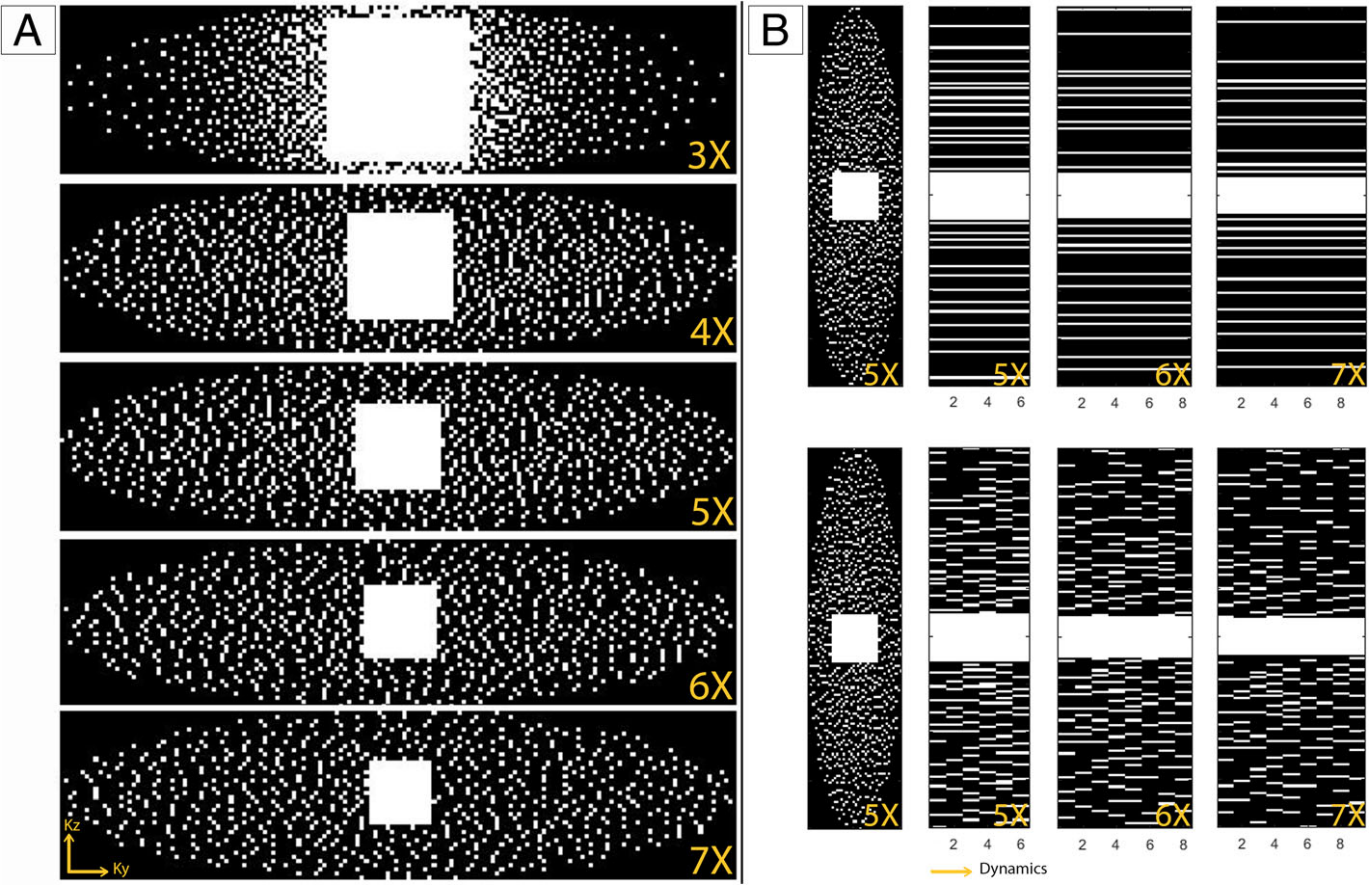
**a** The non-uniform sampling patterns for an undersampling of 3×, 4×, 5×, 6×, and 7× used in the static experiment, consisting of a fully sampled auto calibration area in the centre of k-space and a Poisson disk distribution in the periphery. **b** Similar sampling patterns for an undersampling of 5×, 6×, and 7× used in the dynamic experiment. Top figures show an equal sampling pattern in time, bottom figures show a varying sampling pattern in time

Figure 1a shows the undersampling patterns of the CS-PI-accelerated static scans for all acceleration factors used. Note that these are 2D sampling patterns that were used to accelerate 3D scans in k-*y* and k-*z* phase encoding directions. They are characterised by a non-uniform sampling pattern consisting of a fully sampled auto calibration area in the centre of k-space and a Poisson disk distribution in the periphery. This permits auto-calibrated PI and leads to incoherent aliasing to support CS [9]. In addition to the accelerated static scans, the fully acquired static dataset was also retrospectively undersampled using the same sampling patterns. Retrospective undersampling ensured that the bowel position was identical to the fully sampled scan, allowing determination of a quantitative structural similarity index between the full and accelerated scans. An overview of all reconstructions is given in Table 3.

**Table 3:** Schematic overview of undersampling and reconstruction pipeline of retrospective undersampled and prospective undersampled experiments

Experiment	Acquired k-space	Undersampling preparations	Reconstruction technique
Retrospective	Fully sampled	Non-uniform undersampled 3× to 7×	CS-PI
		Uniform undersampled 3× to 7×	SENSE
Prospective	Non-uniform undersampled	–	CS-PI
	Uniform undersampled	–	SENSE

*CS-PI* compressed sensing and parallel imaging, *SENSE* sensitivity encoding.

#### Dynamic scan sessions

For prospectively accelerated dynamic bowel motility scans, both the use of equal (Fig. 1b, top) and varying (Fig. 1b, bottom) sampling patterns were investigated. The latter is thought to create incoherent sampling over time and allows to exploit sparsity in the temporal domain during CS-PI reconstruction.

For both static and dynamic SENSE-accelerated scans, equidistant (uniform) undersampling patterns in k-*y* and k-*z* directions were used, supplemented by an elliptical k-space shutter. Because of the smaller dimension of the slice encoding direction, a smaller acceleration factor was used in k-*z* direction. All scans and their corresponding acquisition time are listed in Table 4.

**Table 4:** Acceleration settings of the sampling trajectories for CS-PI and SENSE reconstructions

Temporal resolution (s)	CS-PI acceleration factor (×)	SENSE acceleration factor (k-*y* phase encoding direction)	SENSE acceleration factor (k-*z* phase encoding direction)
16.2	1	1	1
5.4	3	2.35	1
4.1	4	3.15	1
3.2	5	4.1	1
2.7	6	4.5	1.1
2.3	7	4.5	1.25

*CS-PI* compressed sensing and parallel imaging, *SENSE* sensitivity encoding. The scan time indicates the temporal resolution for the dynamically acquired scans

### Image reconstruction

Reconstructions were performed offline with a Windows PC in MATLAB (The Mathworks Inc., NA, MA, USA), using an in-house built reconstruction pipeline developed within ReconFrame (GyroTools, Zürich, Switzerland), in combination with the open-source Berkeley Advanced Reconstruction Toolbox (BART) [16], enabling CS-PI reconstruction. For the static scan session, CS-PI-accelerated data was reconstructed with an iterative CS-PI reconstruction technique using L1 wavelet regularisation, as well as total variation regularisation, both in k-*y* and k-*z* dimension. The regularisation weights were optimised for every acceleration factor separately: *λ* = 0.002 (3×, 4×); *λ* = 0.0035 (5×); *λ* = 0.001 (6×); *λ* = 0.005 (7×). For the dynamic scans sessions, CS-PI-accelerated data was reconstructed with an iterative CS-PI reconstruction technique using total variation regularisation in the temporal domain only (regularisation parameter *λ* = 0.01, 20 iterations for all scans). All SENSE-accelerated data was reconstructed using a standard SENSE algorithm available in ReconFrame.

### Evaluation

The reconstruction errors of the retrospectively undersampled scans were evaluated with the mean structural similarity index measure (SSIM), which is a similarity metric designed to predict human visual perception [17]. This metric could not be used in the prospectively undersampled dataset because the metric cannot differentiate between the errors originating from the reconstruction or from possible bowel motility.

Fully sampled, retrospectively and prospectively undersampled CS-PI and SENSE reconstructions were scored by two independent abdominal radiologists with 2 and 20 years’ experience in two sessions. Image quality characteristics (artefacts, contrast, and sharpness) were evaluated. During scoring, the radiologists first assessed the *image quality* characteristics in the scans per set by putting the three images in their preference order for all characteristics separately and for a general quality score assigned accordingly. The score ranged from 1 (least favourable) to 3 (most favourable); an equal score was allowed in the case of similar quality. Secondly, the radiologists scored the *diagnostic quality* of the separate scans within the set with a 4-point scale, as follows: 0 = non-diagnostic; 1 = poor; 2 = adequate; 3 = good. Considering the small sample size of this explorative study, all outcomes were assessed by descriptive statistics only.

#### Static scoring session

In the static experiment scoring session (session 1), CS-PI and SENSE reconstruction were compared to the fully sampled (reference) reconstructions. The radiologists viewed the three blinded scan types as a set (full/CS-PI/SENSE) in a randomised order (Fig. 5), totalling 30 sets (15 prospective undersampled, 15 retrospective undersampled).

#### Dynamic scoring session

In the dynamic experiment scoring session (session 2), CS-PI reconstructions from data acquired with equal sampling pattern (CS1) were compared to those acquired with a varying sampling pattern (CS2) and the SENSE reconstructions. The radiologists viewed the three blinded scan types as a set (CS1, CS2, SENSE), in a randomised order, totalling nine sets, all prospectively undersampled.

## Results

### Static experiment

**Fig. 2:**
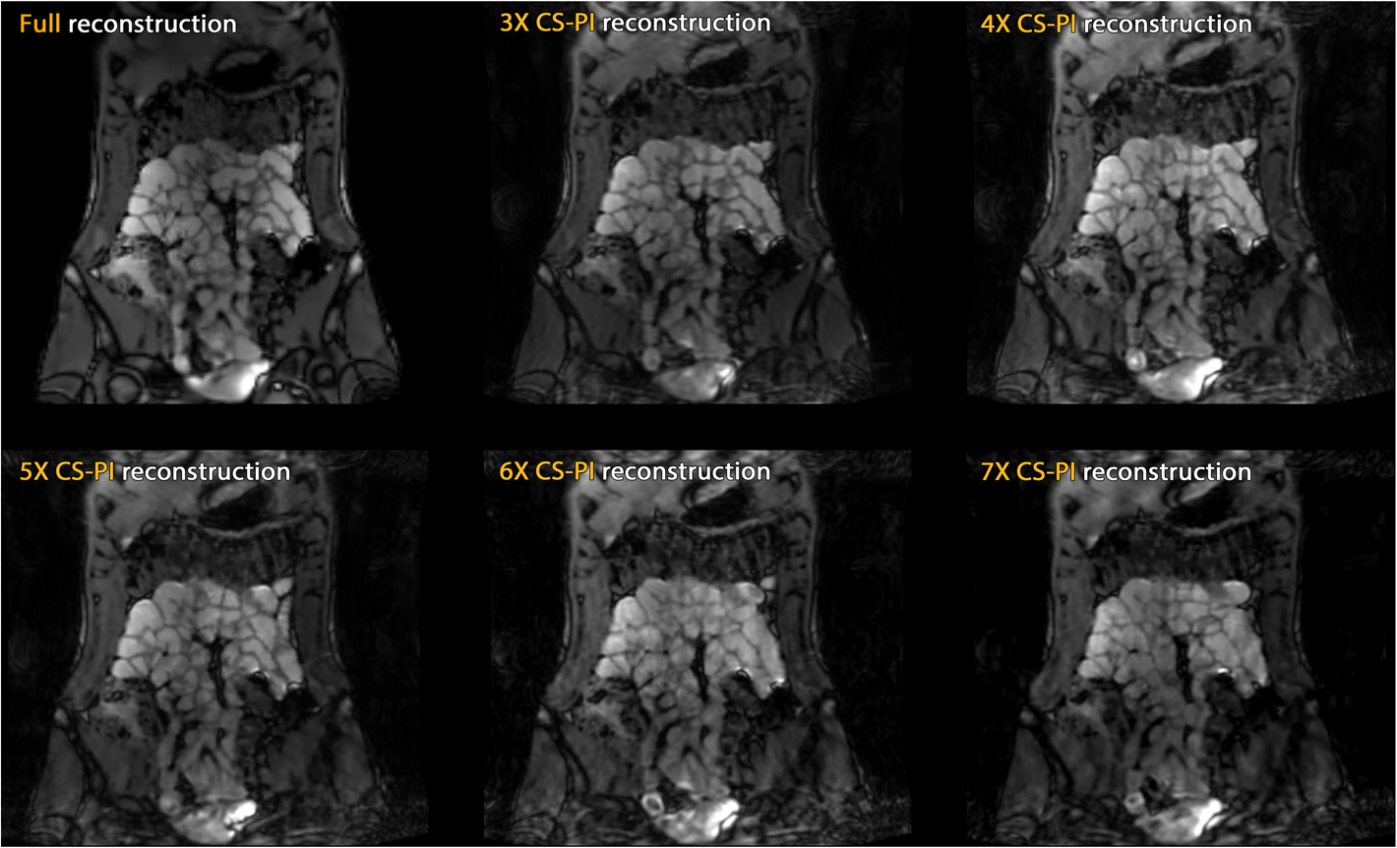
Compressed sensing and parallel imaging (CS-PI) reconstructions from 3×, 4×, 5×, 6×, and 7× accelerated acquisitions (static experiment)

**Fig. 3:**
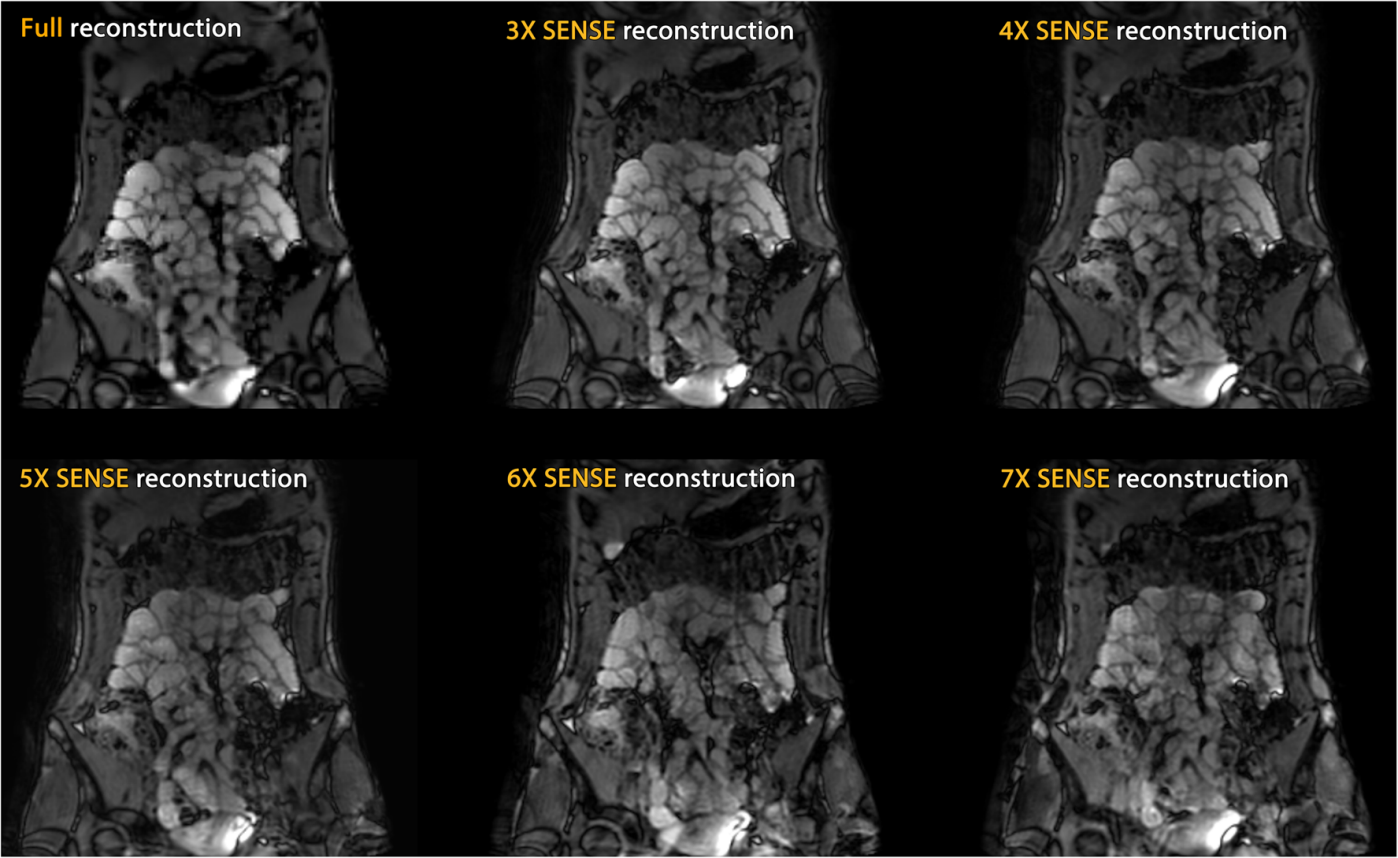
Sensitivity encoding (SENSE) reconstructions from 3×, 4×, 5×, 6×, and 7× accelerated acquisitions (static experiment)

Figures 2 and 3 show reconstructions of the same slice from the static scan session for CS-PI and SENSE data, respectively. Reconstructions were based on fully acquired data, as well as the accelerated 3D scans, from 3× to 7×. Image quality of the fully sampled reference scans showed no motion artefacts.

#### Quantitative evaluation

**Fig. 4:**
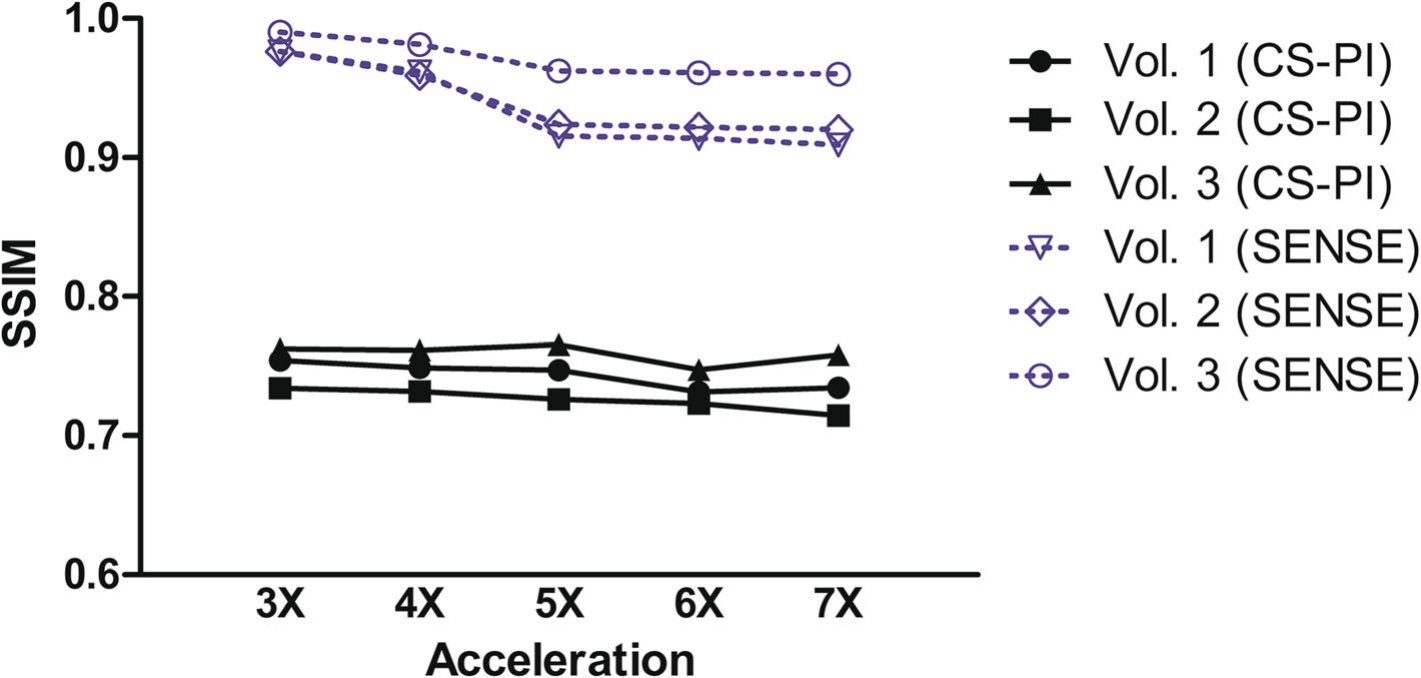
The extent of similarity between the reference scan and the retrospectively undersampled scans, in the static experiment, expressed in the mean structural similarity index measure (SSIM). The mean SSIM of the compressed sensing and parallel imaging (CS-PI) reconstructions is represented with solid lines, the mean SSIM of the sensitivity encoding (SENSE) reconstructions with dotted lines

The mean SSIM values, shown in Fig. 4, represent the extent of similarity between the retrospectively accelerated scans and the fully acquired scan. SENSE reconstructions outperformed the CS-PI reconstructions for every acceleration factor. Surprisingly, mean SSIM values barely differed between examined acceleration factors, in both SENSE and CS-PI.

#### Qualitative (visual) evaluation

**Fig. 5:**
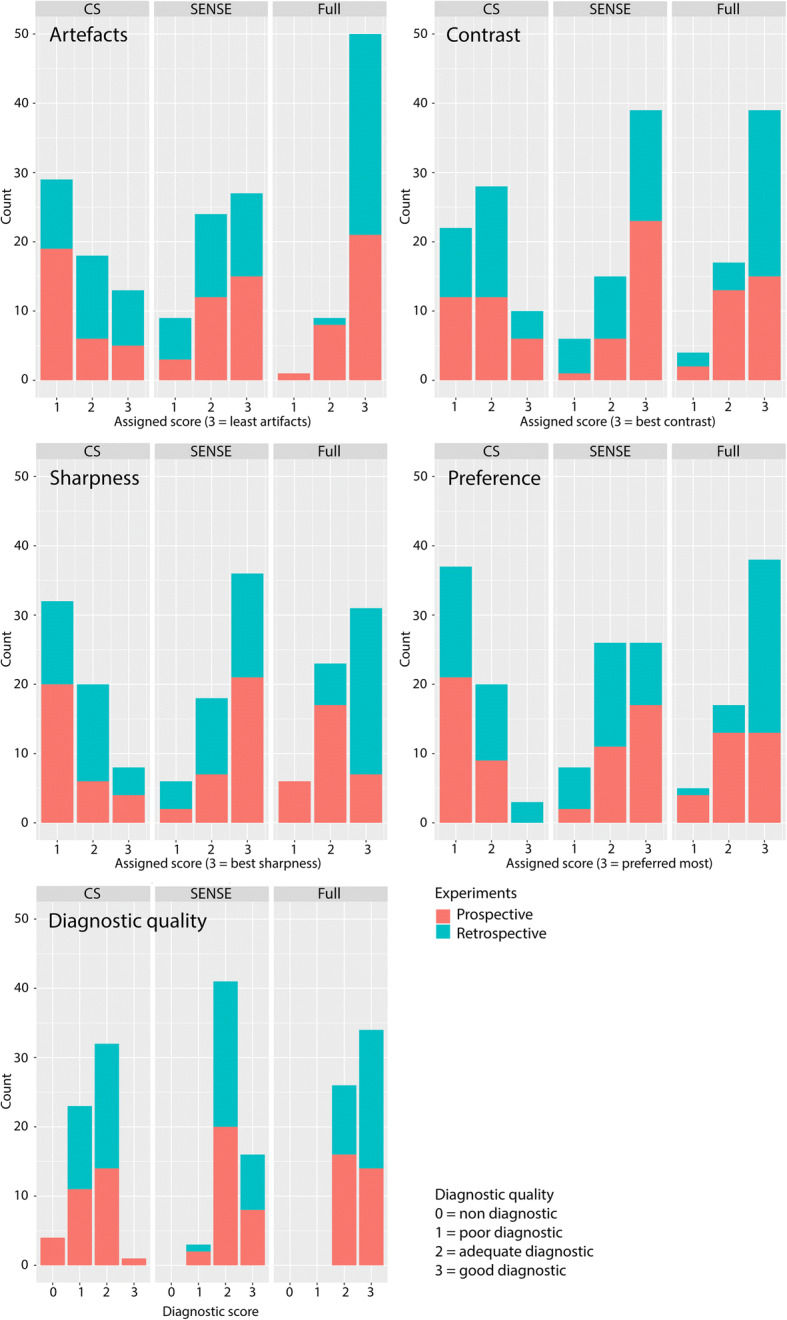
Histograms of static experiment representing the distribution of the prospective and retrospective datasets per scored value among the various scoring variables. *CS* compressed sensing, *SENSE* sensitivity encoding, *Full* fully sampled three-dimensional balanced fast field-echo

Both readers were consistent in assigning scores. There were no substantial differences between the two readers except for the diagnostic quality score of the fully sampled reference scans. Twenty-five out of 30 reference scans were assigned a diagnostic score of 2 (adequate) by reader 1; the other five were assigned a score of 3 (good), whereas reader 2 assigned 29 out of 30 a diagnostic score of 3 (good). Scores regarding image quality of the prospectively undersampled scans obtained by visual assessment were largely similar to the scores from their retrospective counterparts (Fig. 5).

**Fig. 6:**
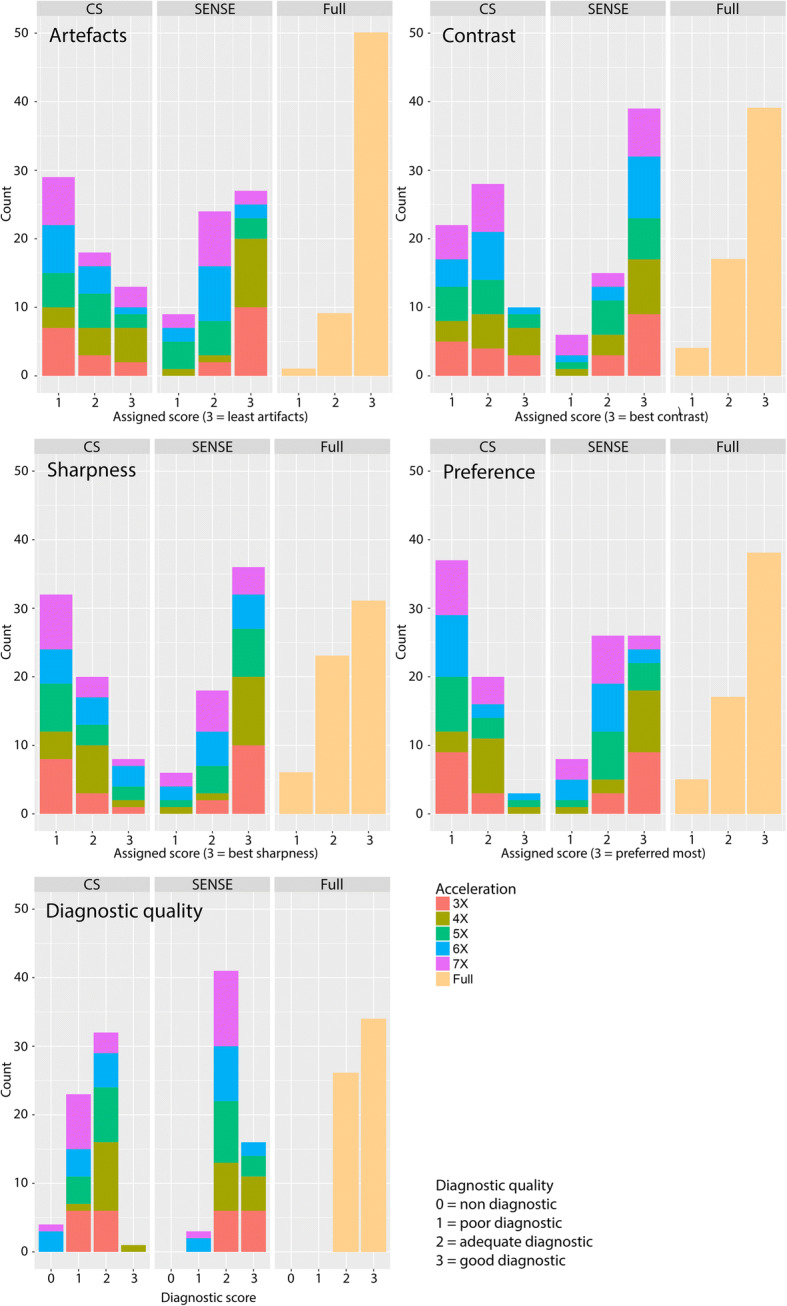
Histograms of static experiment representing the distribution of the accelerated datasets per scored value among the various scoring variables. *CS* compressed sensing, *SENSE* sensitivity encoding, *Full* fully sampled three-dimensional balanced fast field-echo

Compared to the SENSE and reference scan, CS-PI always underperformed in terms of artefacts, contrast, and sharpness, in both the retrospective as well as the prospective undersampled datasets (Figs. 5 and 6). The CS-PI scan was never the preferred scan in the prospective dataset and only three times in the retrospective scans. Concerning diagnostic quality, SENSE-accelerated scans were assigned similar scores as fully sampled scans, with 95% of the SENSE and 100% of the fully sampled scans being assigned a score of 2 or 3 (adequate or good). In contrast, only 55% of the CS-PI scans were assigned a score of 2 or 3, of which only 1.7% corresponded to a score of 3 (good). The 6× and 7× accelerated CS-PI scans were four times assigned with a score of 0 (non-diagnostic), and reconstructions were only once assigned a score of 3 (good). The acceleration composition of the histogram bars in Fig. 6 shows that the scored characteristics were not specifically affected by the acceleration factor.

**Fig. 7:**
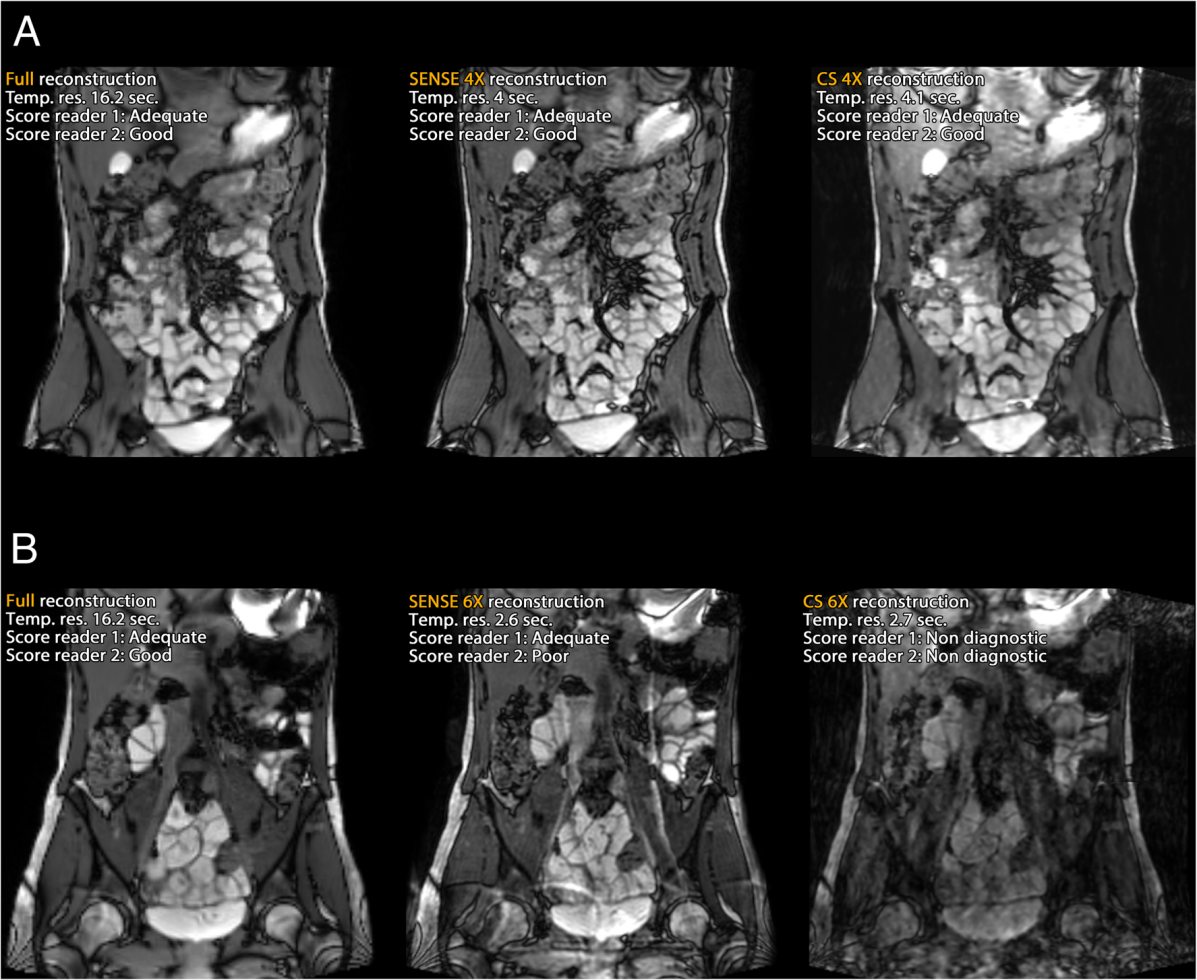
Two scored datasets showing fully sampled three-dimensional balanced fast field-echo reconstructed data (left), sensitivity encoding (SENSE) reconstructed data (middle) and compressed sensing and parallel imaging (CS-PI) reconstructed data (right). The upper set (**a**) was 4× accelerated. All scans were scored adequate or good for diagnostic purpose. The lower set (**b**) was 6× accelerated; in this case, CS-PI reconstruction was scored as non-diagnostic

Figure 7 shows two scored datasets containing reconstructions of fully sampled scans and prospectively undersampled scans. Figure 7a shows representative scans from prospectively 4× accelerated SENSE and CS-PI scans. All scans in this figure were scored in the range of 2 or 3 (adequate or good) for diagnostic purpose. Figure 7b shows representative scans from prospectively 6× accelerated SENSE and CS-PI scans in another volunteer, where the CS-PI scan was assigned a non-diagnostic score.

### Dynamic experiment

**Fig. 8:**
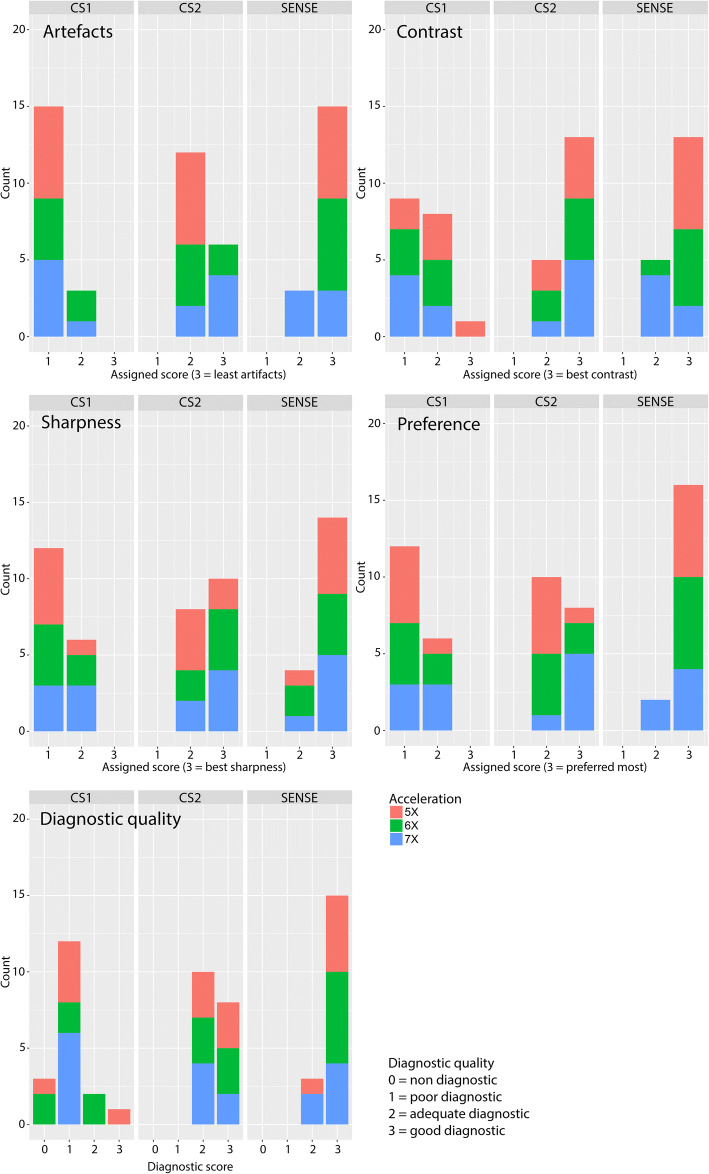
Histograms of dynamic experiment representing the distribution of the accelerated datasets per scored value among the various scoring variables. *CS1*, compressed sensing and parallel imaging (CS-PI) with equal sampling pattern. *CS2*, CS-PI with varying sampling pattern

**Fig. 9:**
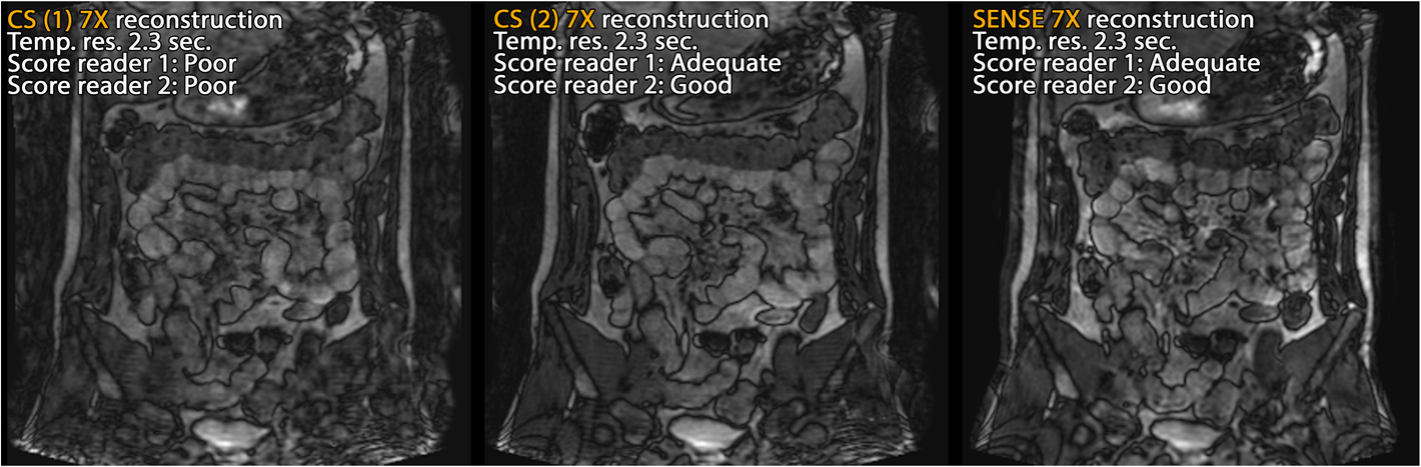
Reconstructions from a representative time frame of a 7× accelerated dynamic acquisitions (dynamic experiment). Left: compressed sensing and parallel imaging (CS-PI) with equal sampling pattern (CS1). Middle: CS-PI with varying sampling pattern (CS2). Right: sensitivity encoding (SENSE)

Although SENSE reconstructions were predominately scored best for (least) artefacts, sharpness, and preference, CS-PI reconstructions acquired with varying sampling patterns performed only slightly less than SENSE on artefacts and sharpness and performed equally well on contrast (Fig. 8). CS-PI with varying sampling pattern performed even somewhat better than SENSE using high (7×) acceleration (on artefacts, contrast, and preference). However, this was not clearly reflected by the diagnostic quality scores. Although all SENSE and CS-PI reconstructions acquired with varying sampling pattern were assigned a diagnostic quality score in the range of 2 or 3 (adequate or good), the distribution of the two scores varied between the acceleration techniques. For the CS-PI scan, 44% was assigned a score of 2 (adequate) and 55% was assigned a score of 3 (good), whereas for the SENSE scans only 17% was assigned a score of 2 (adequate) and the rest, 83%, was assigned a score of 3 (good). The CS-PI with equal sampling pattern underperformed overall on scored image quality characteristics, as well as on scores for diagnostic quality. Figure 9 shows a representative timeframe of a 7× accelerated dynamic dataset, for which only the CS-PI scan with equal sampling pattern was assigned a poor diagnostic score.

## Discussion

This study demonstrates the feasibility of CS-PI in 3D abdominal imaging and presents a comparison of its image quality to a fully sampled scan and conventional SENSE undersampled scans for acceleration factors ranging from 3× to 7×. When static bowel scans were performed, SENSE clearly outperformed CS-PI. Varying sampling patterns for the CS-PI dynamic acquisitions resulted in an image quality comparable to those acquired with SENSE.

When only static volumes were acquired, CS-PI was not able to reconstruct the data with better image quality compared to SENSE. Interestingly, both the quantitative and qualitative evaluations indicated that the acceleration factor itself did not correlate substantially with image quality, for both SENSE and CS-PI. However, in the case of volumes acquired dynamically with varying sampling pattern, the performance of CS-PI increased. Here the incorporation of the sparsity in time seems to add performance to the CS, in line with previous application of CS in the time domain [18]. In contrast to the static experiment, the acceleration factor influenced image quality in the dynamic experiment. SENSE performed better than CS-PI (varying sampling pattern) at an acceleration factor of 5× (5 out of 5 categories), but CS-PI (varying sampling pattern) performed better than SENSE at the highest acceleration factor of 7× (3 out of 5 categories). This indicates that the SENSE techniques had reached a limit.

In this study, sampling trajectories were chosen based on existing literature and freely available reconstruction code. An interesting option that could improve image quality is the use of radial or spiral trajectories in combination with CS. Radial and spiral trajectories are however prone to phase errors due to eddy currents [18], and they both need more sampling time compared to Cartesian sampling, the latter being a limitation for the application explored in this study due to the high temporal resolutions required.

Other researchers have also compared reconstructions with CS-PI to reconstructions with PI alone. For instance, Hollingsworth et al. [19] used CS-PI to measure fat fraction maps and showed CS-PI to be superior to conventional PI. Our results in the static experiment are to some extent in disagreement with the work presented by Hollingsworth et al. [19]. Comparing their data to ours, we note that in their application, breakthrough of incoherent noise at high acceleration factors was avoided by allowing some smoothing of the data. Since for our application the visualisation of the bowel wall was critical, too much smoothing could not be accepted because it would obscure important features of bowel motility.

Regarding the evaluation of the performance of CS-PI, the experience of the observers has to be considered. In general, radiologists are used to evaluate SENSE reconstructions and have no experience with CS-PI reconstructed scans yet. We believe that even though the scans were blinded and randomly presented to the radiologists, experience could have influenced ordering of the CS-PI- and SENSE-accelerated scans with respect to artefacts, contrast, sharpness, and general preference. If this is the case, SENSE is in general favoured, even if the differences between CS-PI and SENSE are small.

In this context, we should also consider the type of artefacts that are present in highly accelerated images (see Fig. 7b, where both SENSE and CS-PI exhibited characteristic artefacts, aliasing, and blurring, respectively). During rating sessions, familiarity with artefacts might again unconsciously favour SENSE over CS-PI. These characteristic artefacts appeared less in the dynamic CS-PI scans acquired with a varying sampling pattern (Fig. 9), which is reflected in the artefact scores shown in Fig. 8.

A limitation of this study is the small sample size; therefore, the analysis remained explorative, resulting in descriptive statistics only. However, the described results provided a good indication of the performance of CS-PI versus SENSE for the specific application of bowel motility imaging, but should not be treated as a definite guideline or be generalised to other applications.

In summary, our study showed that 3D bowel MR imaging can be highly accelerated while maintaining adequate image quality. Clinically available SENSE acceleration outperformed CS-PI when accelerating static bowel scans. While compared to static bowel scans, overall CS-PI performance increased for dynamic scans, but did not outperform SENSE for the investigated acceleration factors. This study therefore warrants further advances in CS-PI acquisition and reconstruction techniques to facilitate its use in clinical bowel motility imaging.

## Abbreviations

2D: Two-dimensional
3D: Three-dimensional
CS-PI: Compressed sensing and parallel imaging
MRI: Magnetic resonance imaging
SENSE: Sensitivity encoding
SSIM: Structural similarity index measure
